# Vulnerability and Resilience in the Caribbean Island States; the Role of Connectivity

**DOI:** 10.1007/s11067-021-09533-w

**Published:** 2021-05-27

**Authors:** Edwina E. Pereira, Albert E. Steenge

**Affiliations:** 1 Central Bank of Aruba, Oranjestad, Aruba; 2 University of Groningen, Faculty of Economics and Business, Groningen, the Netherlands

**Keywords:** Economic resilience, Economic vulnerability, Connectivity, Small Island states, Institutions, Political sovereignty, Systemic interest

## Abstract

It is well-known that small states, because of their size, tend to be less endowed with natural resources than big ones. This makes small states vulnerable and raises the question if specific policies can be implemented to offset the drawbacks of their small size and to increase resilience. We address this question in this paper, thereby focusing on the role of connectivity – between states, organisations, parties, or otherwise – in understanding a country’s vulnerability and resilience. Here ‘policies’ are interpreted as ‘institutions’ in the sense of Douglass C. North (1990), i.e. as ‘humanly devised constraints that structure political, economic and social interaction’. We focus on the Caribbean area, which is characterised by a wide variety of small states, each with its own set of rules and regulations. Within this area, we concentrate on the relationship between three Dutch Caribbean islands, i.e., Aruba, Curaçao, and Sint Maarten, on the one hand, and the Netherlands, the former colonizer, on the other hand. As a first step we have measured the economic vulnerability and resilience of 17 Caribbean island states, both dependent and independent, employing the theoretical framework proposed by Lino Briguglio. The outcomes show that the three Dutch island states are performing comparatively well, although there are individual differences. We provide a first effort to explain this outcome in terms of the continuing interest of the three island states to keep their ties to the former colonizer viable. Here the presence of ‘systemic interest’ as shown by the stakeholders appears to be a most important variable.

**Supplementary Information:**

The online version contains supplementary material available at 10.1007/s11067-021-09533-w.

## Introduction

In this paper we examine the economic vulnerability and economic resilience of Caribbean small island states. In this context, economic vulnerability refers to the exposure of the economy to exogenous shocks, while economic resilience refers to the policy-induced ability of an economy to withstand or recover from the effects of such shocks (Briguglio et al. 2009).

Small states,^1^ and particularly small island states, are characterized by a high degree of economic openness. Economic openness is generally understood to be the degree to which non-domestic actors can or do participate in an economy (Gräbner et al. 2018); One can think here of trade openness and financial openness. Economic openness entails that small states do have economic linkages with other countries. On the one hand, this economic connectivity could be an asset, depending on the quality of the connections. It may provide specific benefits for these small states, such as offering them a worldwide marketplace for their products (Kolb 2008) and knowledge spillovers from their connected countries (Gould et al. 2018). Consequently, economic connectivity could contribute to the economic resilience of these small states. On the other hand, connectivity could imply a high dependence on external economic conditions, thereby increasing the economic vulnerability of small states.

In general, small states are considered to suffer from economic disadvantages related to their size. These size constraints are largely related to a lack of variety of human and natural resources and the small size of their domestic markets (Pereira 2018, p. 18). However, several empirical studies suggest that small open economies and/or small islands do not necessarily suffer from their size constraints (e.g., Easterly and Kraay 2000; Armstrong and Read 2004; Eclac 2001). In fact, the phenomenon that some small island states, like Singapore, Cyprus and Malta, have high economic growth rates and high GDP per capita is referred to as the ‘Singapore Paradox’ by Briguglio (2003). Here the literature points to the role of ‘institutions’, which are understood as the humanly devised constraints that shape human interaction or in other words the ‘rules of the game’ in a society (North 1990, p. 3). They are created by humans to reduce uncertainty and control their environment (Ménard and Shirley 2008, p. 1). Several studies show that small states may have relatively strong institutions which account for their better economic performance (e.g., Bräutigam and Woolcock 2001; Fors 2007).

In fact, one may put forward that, considering their economic openness and the ensuing economic vulnerability, small states need institutions that promote economic resilience (Farrugia 2007). Briguglio (2014) contends that “*small states can succeed economically in spite of their economic vulnerability if they adopt good economic, social, political and environmental governance, which could enable them to reduce and even withstand the negative effects of external shocks*” (p. 58). Strong institutions are thus important for building economic resilience, especially in small states. Therefore, we also explore the relationship between institutions and economic resilience in this paper, since we may expect those countries to perform well that have made institutional choices that mitigate the consequences of their size. However, not much is known about the conditions that are necessary for small states to be able to adopt the set of rules that make up good governance. Here a number of factors may be involved where in particular the role of ‘connectivity’, understood as the set of bonds and linkages between states, organisations or parties, may be important (Caschili et al. 2015; Modica and Reggiani 2015; Östh et al. 2015; Reggiani et al. 2015; Gould et al. 2018). In this paper we focus on the role of the connections between the Caribbean island states and the former colonizers. We concentrate on the relationship between the three Dutch Caribbean islands, i.e., Aruba, Curaçao, and Sint Maarten, on the one hand, and the Netherlands, on the other hand. Particularly, we explore the relationship between political sovereignty (i.e., an institutional choice) and economic resilience by focusing on sovereign as well as dependent Caribbean small island states.

In the present study, we thereby build further on the research by Pereira (2018) who applies the economic vulnerability and resilience framework of Briguglio (2014) to selected Caribbean small island states. Our study similarly focuses on the Caribbean *small* island states. We use two criteria to select these Caribbean small island states, namely the size of these countries and territories and their location in what may be called the Caribbean area. We note that, following the Commonwealth Secretariat – World Bank Joint Task Force on Small States (2000), we define a small state in this study as a country or territory with a population of 1.5 million or less. Small island states are spread around the globe, mostly in the Pacific region and the Caribbean region. Although these small island states have many common characteristics associated with their size, they often have, among other things, substantial cultural, historical, geographical, and economic differences (Pereira 2018, p. 2). According to Fairbairn and Worrell (1996), the Pacific small island states are more geographically isolated from their neighbors compared to the Caribbean small island states which are more closely aligned to each other, and the Caribbean islands are close to the world’s largest markets compared to the Pacific island states which are remote from major markets. Besides, there is a substantial cultural diversity among the Pacific small island states compared to more cultural homogeneity particularly in the English-speaking Caribbean. Our choice to focus exclusively on the Caribbean small island states relates to our aim of having an as homogenous as possible group of small island states to explore the relationship between institutions and economic resilience. This approach substantially reduces the ‘noise’ associated with non-institutional factors for explaining economic resilience such as cultural factors.

Moreover, we select the Caribbean small island states by looking at the Caribbean from both a geographical and socio-historical perspective. Therefore, it includes, besides the islands in the Caribbean Sea or with at least one coast facing the Caribbean Sea, also islands in the Atlantic Ocean (such as The Bahamas and Barbados). Besides these islands, also the mainland states Belize, Guyana, and Suriname are considered as pertaining to the Caribbean. They have many commonalities with the Caribbean such as their legacy of slavery and the plantation system and they have close ties with the Caribbean islands. Illustrative hereof is the fact that these countries are members of the Caribbean Community (CARICOM), which is an organization of Caribbean countries with the objectives of promoting economic integration, foreign policy coordination, human and social development, and security (Pereira 2018, p. 29). Because a great part of their population is settled on the coast, they have characteristics of islands. For instance, Worrell (1993) remarks that “*Belize and Guyana may be considered islands in a vast uninhabited hinterland – most of their populations are settled on the coast*” (p. 190). Pereira (2018) argues that the same applies to Suriname, which had about 87% of its population settled on the coast in 2012 (p. 29). These three countries are considered as small-island developing states by the Small Islands Developing States Network. In this paper, they are referred to as small island states. Table 1 shows some selected indicators for the selected Caribbean small island states.

**Table 1:** Selected indicators of Caribbean small island states for the year 2017

	Population (×1000)	Surface area (km^2^)	Political sovereignty	GDP (million US$)	GDP per capita (US$)	WGI ^a)^
Anguilla	15	91	No	281	18,861	0.90
Antigua and Barbuda	95	442	Yes	1510	14,803	0.51
Aruba	105	180	No	2701	25,655	1.22
Bahamas, The	382	13,940	Yes	11,792	29,825	0.70
Barbados	286	430	Yes	4713	16,494	0.98
Belize	376	22,966	Yes	1902	5077	−0.27
Cayman Islands	63	264	No	4030	65,472	0.85
Curaçao ^b) c)^	160	444	No	3122	19,591	0.76
Dominica	71	751	Yes	497	6719	0.56
Grenada	111	344	Yes	1127	10,451	0.42
Guyana	775	214,969	Yes	3543	4555	−0.24
Saint Kitts and Nevis	52	261	Yes	931	16,818	0.55
Saint Lucia	181	539	Yes	1718	9607	0.60
Saint Vincent and the Grenadines	110	389	Yes	780	7099	0.59
Sint Maarten ^b) c)^	41	34	No	1072	27,116	0.76
Suriname	570	163,820	Yes	3807	6757	−0.14
Trinidad and Tobago	1384	5130	Yes	22,105	16,145	0.12

Source: UNdata; World Bank’s World Development Indicators; CIA World Fact Book; Worldwide Governance Indicators

^a)^Average scores for the six worldwide governance indicators (WGI) for the years 2015–2017

^b)^Data for GDP and GDP per capita refer to the year 2016

^c)^The database of the WGI contains data for the Netherlands Antilles up to the year 2013. Since the latter country was dissolved on October 10, 2010, our assumption is that the data of the Netherlands Antilles refer to both Curaçao and Sint Maarten which used to be part of the Netherlands Antilles prior to that date. Therefore, we take the average of the last three years for which data are available, i.e., 2011–2013

As already mentioned, compared to advanced economies, literature on small states and especially small island states, is relatively scarce, in particular literature related to measuring economic vulnerability and resilience. Lino Briguglio is one of the few authors who has presented an operationalization for the concepts of economic vulnerability and resilience that we shall use as a point of departure. His research shows that small sovereign countries are economically more vulnerable than large sovereign states. In addition, his research illustrates that some of these small sovereign states have built up their economic resilience to counteract the negative effects of their economic vulnerability. The present study differs in several ways from that of Briguglio. The two main differences are the following. First, the aim of this study is to examine how similar small countries sharing common economic disadvantages have dealt with their economic vulnerability, while Briguglio’s study compares countries of all sizes and characteristics with each other, i.e. a much more diverse set of countries. Our results indicate that some small countries are better equipped to deal with exogenous economic shocks than others, possibly because of their institutional choices which have nurtured their economic resilience. Second, because this study focuses on both dependent and sovereign states, we can explore the relationship between political sovereignty (i.e., an institutional choice) and economic resilience. This aspect is not examined by Briguglio, since his research refers only to sovereign states. Our results suggest that there is a statistically significant negative relationship between political sovereignty and economic resilience. This aspect is further explored by focusing on the *socio-political* dimension of vulnerability and resilience in Section 6. We thereby follow Cardinale (2019) in asking attention for the normative aspects involved. In this context Cardinale proposes a role for the concept of ‘systemic interest’, i.e. the interest of parties and stakeholders to keep the system viable. We discuss specific aspects of this concept on the basis of selected episodes in the recent past of the Dutch Caribbean dependent small islands.

This paper is organized as follows. Section 2 elaborates on the consequences of connectivity for the economic vulnerability and economic resilience of small island states. Next, section 3 discusses the relationship between institutions and economic resilience in these small island states. Section 4 reviews the economic vulnerability and resilience framework for small states developed by Briguglio (2014). Section 5 applies this framework for a number of selected Caribbean small island states. Section 6 looks at the role of connectivity from a system interest point of view. Subsequently, section 7 presents concluding remarks.

## The Implications of Connectivity for Economic Vulnerability and Resilience

In this section, we elaborate briefly on the consequences of patterns of connectivity for the economic vulnerability and economic resilience of small island states. A definition of connectivity, which is relevant in this context, is that connectivity refers to a country’s ability to effectively connect to others within a particular network (Arvis and Shepherd 2013). In this paper, we explore two aspects of connectivity, i.e., an economic aspect and a political dimension.

When talking specifically about the economic aspect, we refer to the economic linkages among countries (Gould et al. 2018). Economic relationships among states include trade, business activities, financial relationships, human mobility, and state-sponsored economic relations (Abeldinova and Kemp 2016). Small states, and particularly small island states, are characterized by a high degree of economic openness. They are highly dependent on foreign trade. Because of their small domestic markets, they have to rely on foreign markets for their products (Kuznets 1960, p. 17). Also, they are highly dependent on foreign markets for the imports of products, due to the fact that the domestic demand for products is more broadly than the domestic produced products (Briguglio 1995). This economic openness entails that these small island states have economic linkages with other countries. This economic connectivity could be an asset for these states, depending on the quality of their connections.

Gould et al. (2018, p. 4) note the following about economic connectivity:

“*Being connected to well-connected countries matters for economic growth, but there is complementarity in the various types of connections that enhances growth as well. Countries can benefit from: (i) multiple types of economic links (such as trade, investment, migration, modern telecommunications, and transport) that underpin the movement of technologies and ideas; but also, (ii) the quality of connections in terms of knowledge spillovers and the indirect connections made through partners that are well connected. These are both aspects of interconnectedness that affect growth and growth spillovers*.”

With respect to the political dimension of connectivity, we refer in this study specifically to the relationship of the Caribbean small island states with their former colonizers, because this defines in part a country’s ability to effectively connect to others within a particular network. The former colonizer is part of the network of these countries. We explore this political dimension of connectivity by looking at whether these islands are politically dependent or not. In this context, we note that several authors argue that there is a negative relationship between political sovereignty and economic development in small economies (e.g., Armstrong and Read 2000; Bertram 2004; Oostindie and Sutton 2006). It seems that political dependence offers certain benefits that might have increased the economic resilience and/or may have reduced the economic vulnerability of small countries.

Therefore, we argue that on the one hand connectivity could contribute to the economic resilience of the Caribbean small island states. On the other hand, this connectivity could imply a high dependence on external economic conditions, especially because often small economies rely on volatile export proceeds from a small number of products and foreign markets (Escaith 2001). This high dependence on external economic conditions increases the economic vulnerability of small states. This depends, however, on the quality of the connections, noting that well-diversified connections could mitigate the severity of shocks, and also on the level of diversifications of the connections, where a low level of connectivity could imply lower economic vulnerability because that might mean a lower number of countries that could transmit a shock (Gould et al. 2018).

We come back to this in section 6, thereby taking a look at the recent history of the Dutch Caribbean island states. Here we explore an interesting link between the notions of ‘connectivity’ and ‘systemic interest’. In a recent study, Cardinale (2019) notes that one might expect that a more interconnected system is more vulnerable to shocks, while such a situation also might be an expression of a system where stakeholders have a strong interest in keeping the system viable. In this context the stakeholders’ interest in keeping the existing bonds viable is expressed in a regular ‘updating’ of the existing agreements. Section 6 provides some additional insights along this line.

## Institutions and Economic Resilience

As indicated above, we recently observe a new interest in the role of institutions as a new type of ‘guardians’ against a potentially dangerous outside world. In fact, strong institutions now regularly are seen as being of key importance in safeguarding the economic resilience of, as in our case, small island states. For example, institutions can play a significant role in building resilience to exogenous factors (Beuermann and Schwartz 2018, p.4). According to Acemoglu et al. (2003), countries with weak institutions are more likely to experience high macroeconomic volatility and economic crises. In other words, given their economic vulnerability to external shocks, small states need institutions that will promote economic resilience (Farrugia 2007). Given the importance of institutions for economic resilience, we explore in this section the role of institutions for building economic resilience.

As mentioned before, institutions refer to the humanly devised constraints that shape human interaction or in other words the ‘rules of the game’ in a society (North 1990, p. 3). They determine as well as limit the choices of individuals. These institutions comprise formal constraints (such as rules, constitutions, laws, and property rights), informal constraints (such as socially sanctioned norms of behavior, taboos, traditions, and self-imposed codes of conduct), and their enforcement characteristics (North 1990, p. 4, 36, 47). While institutions are the rules of the game in a society, the ‘players of the game’ are organizations. We distinguish two types of institutions, i.e., economic and political institutions. Economic institutions perform economic functions and include institutions that establish and protect property rights, institutions that facilitate transactions and institutions that permit economic cooperation and organization (Wiggins and Davis 2006). Political institutions include written constitutions, the political system, electoral rules, and the power and capacity of the state to regulate and govern society (Acemoglu and Robinson 2012, p. 42).

As a country’s resources, such as natural resources and human capital, are fixed in the short to medium term, it is of utmost importance for a country’s prosperity how it deals with its resources (Pereira 2018, p. 1). Institutions help to allocate these resources (Acemoglu et al. 2005). They determine how the economy works and the incentives that motivate people (Acemoglu and Robinson 2012, p. 73). Particularly, inclusive economic institutions are conducive to economic activity, productivity growth, and economic prosperity because they promote participation by the great mass of individuals in economic activities (Acemoglu and Robinson 2012, p. 74–75). Institutions that allow the markets to perform adequately are, among others, property rights, regulatory institutions, institutions for macroeconomic stabilization (i.e., fiscal and monetary policy institutions), institutions for social insurance, and institutions of conflict management (Rodrik 2000). In the words of Acemoglu and Robinson (2012, p. 429-430):

*“Inclusive economic institutions that enforce property rights, create a level playing field, and encourage investments in new technologies and skills are more conducive to economic growth than extractive economic institutions that are structured to extract resources from the many by the few and that fail to protect property rights or provide incentives for economic activity. Inclusive economic institutions are in turn supported by, and support, inclusive political institutions, that is, those that distribute political power widely in a pluralistic manner and are able to achieve some amount of political centralization so as to establish law and order, the foundations of secure property rights, and an inclusive market economy.”*

As mentioned before, several empirical studies show that small states do not necessarily suffer from their economic disadvantages because they have relatively strong institutions which account for their better economic performance compared to larger states. According to Pereira (2018), the institutional choices made and the prevailing institutional quality of the Caribbean small states have played a pivotal role in mitigating their economic disadvantages, thereby contributing to their economic performance (p. 202). Because of their small size, these Caribbean small states are considered to suffer from several economic disadvantages. A few of these characteristics are the inherent features that make them economically vulnerable, such as their limited resources, small domestic market, economic openness, and vulnerability to external shocks. Especially the results of Pereira (2018) related to political sovereignty are relevant in the context of this study. Her results suggest that political dependence in the Caribbean small island states are positively associated with higher income per capita. As mentioned before, the present study suggests that there is a negative relationship between political sovereignty and economic resilience.

**Fig. 1:**
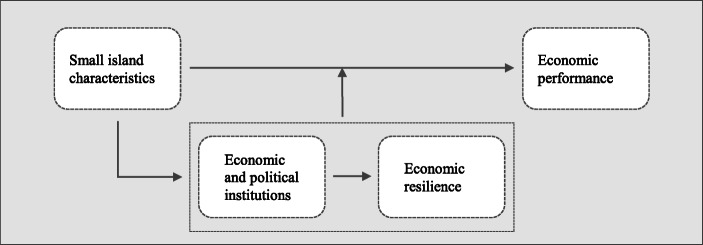
Institutions, economic resilience and economic performance in Caribbean small island states

The framework developed by Pereira (2018) is closely related to the economic vulnerability and resilience framework developed by Briguglio (2014). After all, the concept of economic vulnerability reflects a number of characteristics of small states. This concept is measured by Briguglio (2014) in his economic vulnerability index. The institutions and institutional choices lay the foundation on which policies nurturing economic resilience are made (Pereira 2018, p. 200). These policies are measured in the economic resilience index of Briguglio. We elaborate on these indices in the next section. In Fig. 1, we illustrate explicitly the relationship between institutions and economic resilience. Therefore, we argue that the small size of Caribbean countries has affected their institutional choices, which in turn have impacted their economic resilience. Specifically, institutional quality plays a pivotal role in building economic resilience. Therefore, these institutional choices as well as economic resilience have contributed to mitigate the economic consequences of the small size constraints.

## Briguglio’s Economic Vulnerability and Resilience Framework

Briguglio operationalizes the multi-dimensional concepts of economic vulnerability and economic resilience by estimating two composite indices, i.e., one for economic vulnerability and one for economic resilience for 183 sovereign countries in the world. He applies an equal-weighting approach, assuming that the components of the indices have equal importance. He argues that, as yet, there is not enough objective information to assess the importance of each component to justify the use of unequal weights. Therefore, he opted for the relative simplicity of equal weights.

Briguglio’s economic vulnerability index (EVI) captures several inherent characteristics of small island states which are related to their size. It covers the following four components: (i) trade openness, (ii) export concentration, (iii) dependence on strategic imports, and (iv) proneness to natural disasters (Briguglio 2014). Due to their limited resources and small domestic market, small nations tend to concentrate their economic production on a limited range of activities (e.g., Kuznets 1960). However, consumers and producers demand more types of products than those domestically produced, which results in the need to import foreign products. To pay for their import bill, these small nations are dependent on export receipts (Briguglio 1995). Consequently, small island states have a heavy reliance on foreign trade and are therefore characterized by a high degree of trade openness that renders them vulnerable to economic conditions in the rest of the world. This economic vulnerability is exacerbated when a country is dependent on a few export products (Briguglio 2014) and thus has a high degree of export concentration. The degree of economic vulnerability can be increased further when a country depends also on imports of essential products (i.e., strategic imports) which are price and income inelastic (Briguglio 2014). This implies that an increase in the price of these products and a decrease in income lead to a smaller decrease in the demand for these products. Finally, small states have a high economic vulnerability related to their susceptibility to natural disasters which may affect the entire population and economy (Commonwealth Secretariat – World Bank Joint Task Force on Small States 2000).

Briguglio’s economic resilience index (ERI) concerns policy-induced measures aimed at reducing economic vulnerability. These measures include macroeconomic stability, market flexibility, and good political, social, and environmental governance. He associates macroeconomic stability with a situation in which a country has room for maneuver in the event of an adverse external shock; such a situation is encountered when a country has a sustainable fiscal position, low inflation, and an external balance as reflected in the current account position. Market flexibility makes a country more resilient to economic shocks, because it allows a country to reallocate resources quickly and effectively following an economic shock as a consequence of limited regulatory constraints and bureaucratic procedures (Briguglio 2014). Briguglio (2014) points out that good political, social, and environmental governance can strengthen an economy’s resilience, because external shocks can be expected to be better absorbed and counteracted in an atmosphere of predictable laws and credible policies, properly developed relations enabling an effective functioning of the economic apparatus without the problem of civil unrest, and environmental law and policy conducive to environmental conservation, protection and use of natural resources.

Briguglio’s ERI, concretely speaking, includes the following three main components: (i) macroeconomic stability, (ii) market flexibility (adjusted for financial safety), and (iii) an index concerning political, social and environmental governance as estimated by three subcomponents, i.e., a political governance index, a social development index, and an environmental management index (Briguglio 2014).

Briguglio (2014) argues that the overall conclusion of his research is that small states tend to be highly exposed to exogenous economic shocks because of their inherent characteristics. Therefore, they should focus on a holistic approach encompassing resilience building social, political, environmental, governance and economic policies.

## Measuring Economic Vulnerability and Resilience in Caribbean Small Island States

Following Briguglio (2014) and Pereira (2018), we estimate the economic vulnerability and economic resilience of 17 Caribbean small island states.^2^ Twelve of these economies are sovereign states and are thus also included in the study of Briguglio. These are Antigua and Barbuda, The Bahamas, Barbados, Belize, Dominica, Grenada, Guyana, Saint Kitts and Nevis, Saint Lucia, Saint Vincent and The Grenadines, Suriname, and Trinidad and Tobago. Five Caribbean islands – Anguilla, Aruba, Cayman Islands, Curaçao, and Sint Maarten – are dependent countries or territories and are, therefore, not part of Briguglio’s study. Anguilla and Cayman Islands are British Overseas Territories, while Aruba, Curaçao, and Sint Maarten are autonomous countries within the Kingdom of the Netherlands. As mentioned before, political sovereignty is an institutional choice which could impact the economic resilience of countries. We have argued in section 3 that institutional choices as well as economic resilience may contribute to mitigate the negative economic consequences of the size constraints of small countries. We note here that several authors argue that there is a negative relationship between political sovereignty and economic development in small economies (e.g., Armstrong and Read 2000; Bertram 2004; Oostindie and Sutton 2006). In line with this, we expect that the distinction between dependent states and sovereign states could be relevant for the concept of economic resilience.

### Methodology

The calculation of the EVI and the ERI comprises two steps. Firstly, we identify outliers for each individual component of the EVI and ERI to reduce their distorting effects. After identifying observation X_i_ as an outlier, its value is capped. Due to small sample size, we use the simple standard deviation method to identify outliers in the data. An observation X_i_ is an outlier in the following case:

$$ \left[\left({\mathrm{X}}_{\mathrm{i}}-\overline{\mathrm{X}}\right)/\mathrm{SD}\right]<-2\ \mathrm{or}\ \left[\left({\mathrm{X}}_{\mathrm{i}}-\overline{\mathrm{X}}\right)/\mathrm{SD}\right]>2 $$

where

SD: standard deviation of n observations.

$\overline{\mathrm{X}}$: average of n observations.

X_i_: observation i in an array of n observations.

After identifying an outlier, its value is capped as follows:

$$ {\displaystyle \begin{array}{c} If\ \left[\ \left({\mathrm{X}}_{\mathrm{i}}-\overline{\mathrm{X}}\right)/\mathrm{SD}\ \right]>2\  Cap\ {\mathrm{X}}_{\mathrm{i}}=\overline{\mathrm{X}}+2\ \mathrm{SD}\\ {} If\ \left[\ \left({\mathrm{X}}_{\mathrm{i}}-\overline{\mathrm{X}}\right)/\mathrm{SD}\ \right]<-2 Cap\ {\mathrm{X}}_{\mathrm{i}}=\overline{\mathrm{X}}-2\ \mathrm{SD}\end{array}} $$

Secondly, the data for the components of the EVI and ERI are rescaled (or normalized) using the Max-Min formula.^3^ Each component of the EVI and ERI can consist of subcomponents. Note that after calculating the composite index based on the weighted averages of the indices of its (sub)components, the composite index is rescaled again. The formulas for EVI and ERI are the same as those of Briguglio (2014) with the exception of data sources in some cases.^4^

Besides estimating the EVI and the ERI for these 17 Caribbean small island states, the present study applies a Mann-Whitney U test,^5^ a nonparametric test, to examine if the ERI scores of the sovereign states are significantly different from those of the dependent countries and territories. This analysis gives insight into the role of political sovereignty in economic resilience. The null hypothesis of the Mann-Whitney U test is that the two independent groups are homogenous and have the same distribution, implying that the medians of the two respective groups are not different. In contrast, the alternative hypothesis is that the distributions of the two groups are different, meaning that the two medians differ. Therefore, the following hypotheses are tested:

*H*_*o*_ : *θx* = *θy.*

*H*_1_ : *θx* ≠ *θy.*

where *θ*_*x*_ is the median of group *x* and *θ*_*y*_, is the median of group *y*. These hypotheses are tested at the *α* = 0.05 significance level.

For each group a U-statistic is calculated, i.e.

$$ {U}_x={n}_x{n}_y+\frac{n_x\left({n}_x+1\right)}{2}\kern0.5em -{R}_{x.} $$

$$ {U}_y={n}_x{n}_y+\frac{n_y\left({n}_y+1\right)}{2}\kern0.5em -{R}_y $$

where *n*_*x*_ is the sample size of group *x*, *n*_*y*_ is the sample size of group *y*, *R*_*x*_ is the sum of the ranks assigned to group *x*, and *R*_*y*_ is the sum of the ranks assigned to group *y*. *H*_*o*_ is rejected if *min* (*U*_*x*_, *U*_*y*_) ≤ U critical value at a significance level of *α* = 0.05.

One major constraint of our study is limited data availability, particularly for the dependent states, because most databases cover only sovereign states. Therefore, we use alternative data sources, mostly from statistical offices and/or central banks of the respective countries as well as International Monetary Fund (IMF) Article IV mission reports of selected countries, and we impute data in case of missing data. Due to limited data availability, we use simple weights for the economic vulnerability and economic resilience indices, because one of the biggest challenges is to get relevant data for all these Caribbean small island states to construct weighted indices.

Another limitation of our study refers to the rather homogenous group of Caribbean small island states. Because of this, one could expect that the degree of vulnerability is quite similar among these small island states. Nevertheless, even between Caribbean small island states there are differences related to their size, their lack of human and natural resources, and their geographical characteristics, which may have resulted in differences in their economic vulnerability.

The small sample size is an additional constraint of our study. As a result, we have used relatively simple statistical tests such as Mann-Whitney U-test instead of using more complex and advanced statistical and econometric tests and techniques. Moreover, our study focuses exclusively on the Caribbean small island states and thus may not be representative of small island states in general.

A final constraint of our study relates to the use of proxies for estimating the components of the economic vulnerability and resilience indices. Consequently, these proxies may not reflect the developments in the respective components as intended. One example hereof is the big difference in the outcomes for the proneness to natural disasters for Anguilla and Sint Maarten which will be illustrated in the calculations of the economic vulnerability index. Although Anguilla is a close neighbor of Sint Maarten, its proneness to natural disasters is much lower. This could be related to our measure of proneness to natural disasters. This variable is proxied by the economic damage (in percent of GDP) caused by natural disasters. The data for this variable are from the EM-DAT database, which is the most widely used in the literature. Limitations of the latter database are that economic damage is reported for only 32% of disasters (36% for small states), while in general, richer countries tend to have better records of economic damages than low income countries (International Monetary Fund 2016). Moreover, this measure of proneness to natural disasters does not reflect exclusively the degree to which a country is vulnerable to natural disasters as a result of its exposure to these events, but also its disaster mitigating measures which may have limited the economic damage. Therefore, although Anguilla and Sint Maarten are close neighbors, there may be other factors influencing their degree of proneness to natural disasters.

The estimated EVI and ERI scores in this section are not fully comparable to those of Briguglio (2014) for a number of reasons. First, by measuring economic vulnerability and resilience exclusively for these 17 Caribbean small countries, our analysis focuses on the differences in the degree of vulnerability and resilience between the selected Caribbean countries, while Briguglio (2014) compares 183 countries of all sizes with each other, i.e. a much more diverse set of countries. Second, we use more recent data than Briguglio (2014). Third, due to a lack of data, alternative data sources and databases are used for estimating (sub)components of the EVI and ERI, while in some cases missing data are imputed. Fourth, our sample includes both dependent states and sovereign states, while the sample of Briguglio (2014) includes only sovereign states. Fifth, we apply a statistical test to determine outliers, while Briguglio puts an arbitrary cap on outliers.

### Results for Economic Vulnerability

Table 2 shows our calculations for the economic vulnerability index (EVI) and its components. Our analysis suggests that Sint Maarten has the highest economic vulnerability score, particularly because of its high proneness to natural disasters. We note that Sint Maarten is the second smallest country with a population of about 41 thousand people. Dominica (population of 71 thousand people) has the second highest vulnerability score, also largely because of its high proneness to natural disasters. We note that both Sint Maarten and Dominica have the highest proneness to natural disasters, much higher than all other selected Caribbean small island states. Aruba (population of 105 thousand people) has the third highest economic vulnerability score, despite the fact that its score for proneness to natural disasters is zero. This outcome is due to its high degree of export concentration, trade openness, and dependence on strategic imports. Guyana is the least vulnerable country with below average scores for all four components of the EVI. We remark that Guyana is the second largest country with a population of 775 thousand people.

**Table 2:** EVI of selected Caribbean small island states ^a)^

Country	OPN	EXN	DSI	DST	EVI	EVI RS
Anguilla	0.530	0.811	0.191	0.222	0.438	0.451
Antigua and Barbuda	0.571	0.846	0.419	0.189	0.506	0.572
Aruba	0.790	0.825	0.693	0.000	0.577	0.698
Bahamas, The	0.000	0.675	0.515	0.089	0.319	0.239
Barbados	0.197	0.186	0.572	0.000	0.239	0.095
Belize	0.445	0.276	0.436	0.119	0.319	0.238
Cayman Islands	0.448	1.000	0.396	0.314	0.540	0.631
Curaçao	0.578	0.281	0.426	0.020	0.326	0.251
Dominica	0.304	0.902	0.447	1.000	0.663	0.851
Grenada	0.361	0.948	0.439	0.342	0.523	0.601
Guyana	0.260	0.000	0.390	0.091	0.185	0.000
Saint Kitts and Nevis	0.382	0.656	0.212	0.298	0.387	0.359
Saint Lucia	0.486	0.712	1.000	0.010	0.552	0.653
Saint Vincent and The Grenadines	0.170	0.627	0.422	0.069	0.322	0.243
Sint Maarten	1.000	0.987	0.000	1.000	0.747	1.000
Suriname	0.209	0.539	0.322	0.000	0.267	0.146
Trinidad and Tobago	0.164	0.184	0.698	0.000	0.262	0.136

Source: Authors’ calculations

^a)^OPN = Trade openness; EXN = Export concentration; DSI = Dependence on strategic imports; DST = Disaster proneness; EVI = Economic vulnerability index; EVI RS = Rescaled economic vulnerability index

**Fig. 2:**
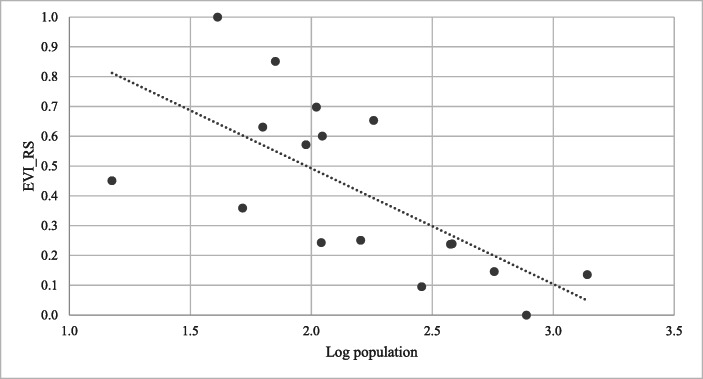
The relationship between population size and economic vulnerability ^a)^ The threshold between high and low EVI scores is 0.421, which is equal to the average EVI scores of the selected countries. BAP countries have a log population of less than 2.449, while AAP countries have a log population of more than 2.449

These results tend to indicate that there might be a negative relationship between population size and the EVI. This relationship is illustrated in Fig. 2. Results of the Spearman rank correlation,^6^ denoted by r_s_, indicate that there is a significant negative correlation between population size and EVI (r_s_ = −0.748, *p* = .001). Thus, there seems to be a negative association between country size as measured by population and the degree of economic vulnerability. Noticeably, the selected Caribbean small island states with an above average population (AAP) size have a below average economic vulnerability score,^7^ while the majority of the selected Caribbean small island states with a below average population (BAP) size have an above average economic vulnerability score. Moreover, a Mann-Whitney U test confirms that there is a negative relationship between population size and EVI. The latter test indicates that the group of countries with BAP has a significantly higher median economic vulnerability (Mdn = 0.601) than the median economic vulnerability (Mdn =0.141) of the group of countries with AAP (see Table 3). Therefore, our results indicate that the smaller the Caribbean island states, the more vulnerable they are.

**Table 3:** Results of the Mann-Whitney U-test for significance of group differences for *EVI* and its components ^a) b) c)^

	BAP	AAP	*min* (*U*_*x*_, *U*_*y*_)	*U critical value*
*n*_*i*_	11	6		
Median of EVI_RS	0.601	0.141	0*	13
Median of OPN	0.486	0.203	7*	13
Median of EXN	0.825	0.231	4*	13
Median of DSI	0.422	0.475	25	13
Median of DST	0.222	0.045	14	13

Source: Authors’ calculations

^a^BAP = group of countries with population below the average population for all 17 selected Caribbean small states; AAP = group of countries with population above the average population for all 17 selected Caribbean small states;

OPN = Trade openness; EXN = Export concentration; DSI = Dependence on strategic imports; DST = Disaster proneness; EVI RS = Rescaled economic vulnerability index.

^b^The BAP group consists of Anguilla, Antigua and Barbuda, Aruba, Cayman Islands, Curaçao, Dominica, Grenada, Saint Kitts and Nevis, Saint Lucia, Saint Vincent and The Grenadines, and Sint Maarten. The AAP group comprises The Bahamas, Barbados, Belize, Guyana, Suriname, and Trinidad and Tobago.

^c^The superscript * denotes significance at the 5% level.

### Results for Economic Resilience

Table 4 shows the economic resilience scores for the selected Caribbean small island states. The results indicate that Cayman Islands (population of 63 thousand people) is the most resilient country, followed by Trinidad and Tobago (population of 1384 thousand people). Remarkably, Aruba has not only the third highest score for economic vulnerability but also for economic resilience. The least resilient country is Suriname (population of 570 thousand people), which has low scores for almost all sub-indices.

**Table 4:** ERI of selected Caribbean small open economies ^a) b)^

Country	STB	MFX	FIN	PGV	SOC	ENV	ERI	ERI RS
Anguilla	0.338	0.517	0.046	0.785	0.454	0.582	0.478	0.525
Antigua and Barbuda	0.125	0.721	0.638	0.521	0.359	0.457	0.436	0.464
Aruba	0.384	0.517	0.966	1.000	0.297	0.510	0.605	0.710
Bahamas, The	0.413	0.563	0.768	0.649	0.467	0.287	0.528	0.598
Barbados	0.014	0.433	0.570	0.836	0.550	0.318	0.394	0.403
Belize	0.231	0.571	0.294	0.000	0.301	0.400	0.283	0.241
Cayman Islands	0.939	0.517	1.000	0.752	1.000	1.000	0.804	1.000
Curaçao	0.196	0.517	0.675	0.691	0.761	0.482	0.474	0.518
Dominica	0.097	0.847	0.614	0.557	0.272	0.465	0.460	0.498
Grenada	0.000	0.272	0.624	0.462	0.430	0.122	0.253	0.197
Guyana	0.137	0.371	0.178	0.022	0.000	0.000	0.158	0.058
Saint Kitts and Nevis	0.042	0.400	0.662	0.549	0.299	0.599	0.341	0.325
Saint Lucia	0.183	1.000	0.288	0.583	0.335	0.335	0.502	0.560
Saint Vincent and the Grenadines	0.062	0.471	0.486	0.576	0.246	0.753	0.362	0.356
Sint Maarten	0.308	0.517	0.675	0.691	0.263	0.400	0.479	0.526
Suriname	0.224	0.000	0.000	0.090	0.167	0.255	0.118	0.000
Trinidad and Tobago	1.000	0.848	0.888	0.263	0.337	0.789	0.740	0.908

Source: Authors’ calculations

^a^STB = Macroeconomic stability; MFX = Market flexibility; FIN = Financial safety; PGV = Political governance; SOC = Social development; ENV = Environmental management; ERI = Economic resilience index; ERI RS = Rescaled economic resilience index.

^b^The market flexibility (adjusted for financial safety) component of the ERI of Briguglio (2014) comprises two subcomponents, i.e., market flexibility and financial safety.

**Fig. 3:**
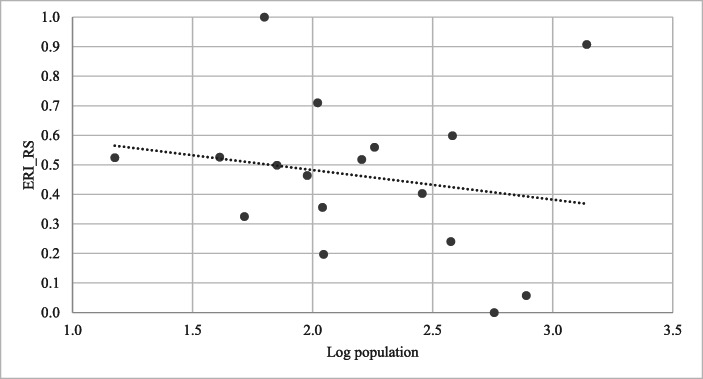
The relationship between population size and economic resilience ^a)^ The threshold between high and low ERI scores is 0.464, which is equal to the average ERI score of selected countries. See also Fig. 2, under a)

Figure 3 shows the relationship between population size and the calculated economic resilience index. Results of the Spearman rank correlation^8^ indicates that there is a weak negative correlation between population size and ERI but this relationship is not significant (r_s_ = −0.225, *p* > .005).^9^ In addition, the results of a Mann-Whitney U test indicate that the economic resilience of the group of smaller countries is not significantly higher than that of the larger countries (see Table 5). Therefore, it seems that the size of the country is not an indicator of the degree of resilience.

**Table 5:** Results of the Mann-Whitney U-test for significance of group differences for *ERI* and its components for country size^a) b) c)^

	BAP	AAP	*min* (*U*_*x*_, *U*_*y*_)	*U critical value*
*n*_*i*_	11	6		
Median of ERI_RS	0.518	0.322	23	13
Median of STB	0.183	0.228	25	13
Median of MFX	0.517	0.498	29	13
Median of FIN	0.638	0.432	24	13
Median of PGV	0.583	0.177	16	13
Median of SOC	0.335	0.319	29	13
Median of ENV	0.482	0.302	16	13

Source: Authors’ calculations

^a)^BAP = group of countries with population below the average population for all 17 selected Caribbean small states; AAP = group of countries with population above the average population for all 17 selected Caribbean small states; STB = Macroeconomic stability; MFX = Market flexibility; FIN = Financial safety; PGV = Political governance; SOC = Social development; ENV = Environmental management; ERI RS = Rescaled economic resilience index

^b)^The BAP group consists of Anguilla, Antigua and Barbuda, Aruba, Cayman Islands, Curaçao, Dominica, Grenada, Saint Kitts and Nevis, Saint Lucia, Saint Vincent and The Grenadines, and Sint Maarten. The AAP group comprises The Bahamas, Barbados, Belize, Guyana, Suriname, and Trinidad and Tobago

^c)^The superscript * denotes significance at the 5% level

Regarding one particular aspect we may look a bit deeper. As mentioned before, political sovereignty may impact the economic resilience of countries. A Mann-Whitney U test indicates that the economic resilience score (Mdn = 0.380) is significantly lower for the group of sovereign countries than for dependent countries (Mdn = 0.526) (see Table 6). Therefore, it seems that there is a significant negative relationship between political sovereignty and economic resilience. This is largely due to significantly lower scores for macroeconomic stability and political governance for the group of sovereign countries than for the group of dependent countries.

**Table 6:** Results of the Mann-Whitney U-test for significance of group differences for *ERI* for political sovereignty ^a) b) c)^

	Political sovereignty	No political sovereignty	*min* (*U*_*x*_, *U*_*y*_)	*U critical value*
*n*_*i*_	12	5		
Median of ERI_RS	0.380	0.526	10*	11
Median of STB	0.131	0.338	11*	11
Median of MFX	0.517	0.517	30	11
Median of FIN	0.592	0.675	15	11
Median of PGV	0.535	0.752	4*	11
Median of SOC	0.318	0.454	19	11
Median of ENV	0.368	0.510	15	11

Source: Authors’ calculations

^a^STB = Macroeconomic stability; MFX = Market flexibility; FIN = Financial safety; PGV = Political governance; SOC = Social development; ENV = Environmental management; ERI RS = Rescaled economic resilience index.

^b^The following countries have political sovereignty: Antigua and Barbuda, The Bahamas, Barbados, Belize, Dominica, Grenada, Guyana, Saint Kitts and Nevis, Saint Lucia, Saint Vincent and The Grenadines, Suriname, and Trinidad and Tobago. Anguilla, Aruba, Cayman Islands, Curaçao, and Sint Maarten are dependent countries or territories.

^c^The superscript * denotes significance at the 5% level.

### Comparing Economic Vulnerability and Economic Resilience

**Fig. 4:**
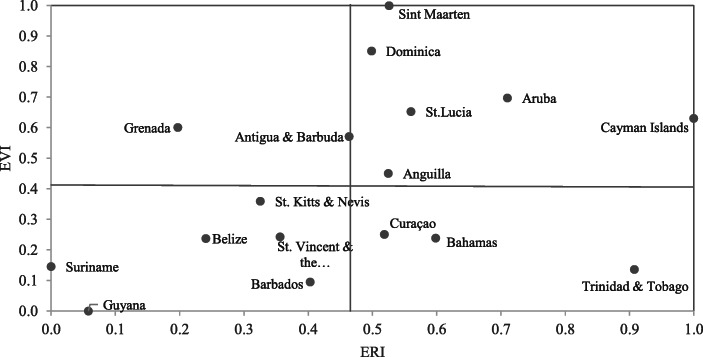
EVI and ERI scores ^a)^ Source: Authors’ calculations ^a)^ The threshold between low and high vulnerability is 0.421, equal to the average vulnerability score of all 17 countries. The threshold between low and high resilience is 0.464, equal to the average resilience score of all 17 states

Figure 4 compares the results of the calculated economic vulnerability and economic resilience scores for the 17 selected Caribbean small island states. This comparison shows that even between the Caribbean small island states there are differences in their degree of economic vulnerability, indicating that the smaller states are even more vulnerable than their larger peers. Noticeably, four of the five dependent states (i.e., Cayman Islands, Aruba, Sint Maarten, and Anguilla) are in the group of states that are highly vulnerable but also highly resilient as compared to the other countries. Our calculations indicate that these dependent countries and territories are among the most vulnerable countries around the world, but with a high resilience, considering that they are in general more vulnerable and resilient than the other sovereign Caribbean small island states that have been classified by Briguglio (2014) as economically successful states. He defines economically successful states as countries that are highly vulnerable (compared to the average EVI of all 183 states included in his study) but have a high economic resilience as compared to the average ERI in his study.

## Systemic Interest in the Dependent Caribbean Small Islands

In the previous section, we have seen that the dependent countries and territories seem to perform above average with respect to economic resilience. In this regard, we note that Pereira (2018) has come to a similar conclusion in her study which consisted largely of the independent Caribbean countries. The only dependent country in her study, i.e. Aruba, also seemed to be the most resilient one. So, how to interpret the above findings? In addition, we have noticed that political sovereignty in the Caribbean small island states appears to be negatively associated with economic resilience. In the following two subsections, this outcome is further explored by looking at the socio-political dimension of economic resilience. Here we introduce the concept of 'systemic interest' to better understand economic resilience as observed in the case of dependent Caribbean countries and territories. We apply this concept to the three Dutch Caribbean islands which are part of the Kingdom of the Netherlands. These islands are performing comparatively well compared to their sovereign peers. We provide a first effort to explain this outcome in terms of the continuing interest of these islands to keep their ties to the Netherlands viable.

### The Concept of Systemic Interest

For a further understanding of what may have happened, we may need additional tools. Vulnerability and resilience have been discussed by a number of authors, from a variety of corners. For instance, Caschili et al. (2015) elaborate on several papers dedicated to the resilience and vulnerability of systems. According to Modica and Reggiani (2015), “*the focus of economic resilience seems, on the one hand to be on analysis of the speed with which a system returns to its pre-shock condition (engineering resilience), and on the other hand on the capacity, of a system to reach new possible equilibria (ecological resilience)*” (p. 215). Also, Reggiani et al. (2015) elaborate on these concepts in the transport context, while Reggiani et al. (2002), e.g., point to the role of evolutionary concepts. However, if we wish to focus on patterns that become visible in the medium or long run, a recent proposal by Cardinale (2019) can provide further insight. In recent work Cardinale asks attention for the socio-political dimension of both vulnerability and resilience. He thereby focuses on the role of connectivity and the normative character involved. In his view, normativity enters in the form of the interests of the stakeholders. The key concept here is ‘systemic interest’. Following Cardinale (2019), we define systemic interest as the interest of stakeholders in keeping the system viable. Cardinale thereby points out that connectivity (through the interconnected flows of commodities and services) *also* may imply that certain policies, say one-sided action by one of the parties involved, can threaten the overall status quo and make the system less viable, thereby jeopardizing the interest of *all* stakeholders. This possibility introduces a normative element in the relation between vulnerability and resilience in the sense that these are not objectives in themselves, but rather are constraints on the behaviour of the stakeholders. Systemic interest, which exists if the parties involved benefit from continuing the existing relation, can lead to actions designed to strengthen their bond. However, there is a catch, in the sense that such policies also can lead to lower flexibility and thereby to increased vulnerability and lower resilience (Cardinale 2019). He then points out that the approach can be extended in several directions. Change over time, for example, may be understood in this way.

In the light of our findings in Section 5, a small excursion to the Dutch Caribbean island states Aruba, Curaçao, and Sint Maarten may be illustrative. The relation between these states and the Netherlands have, recently, undergone important changes. To understand these in terms of ‘systemic interest’, we may use the concept of ‘institutions’ as proposed by Douglass North (1990, 1991). To quote:

“*Institutions are the humanly devised constraints that structure political, economic and social interaction. They consist of both informal constraints (sanctions, taboos, customs, traditions, and codes of conduct), and formal rules (constitutions, laws, property rights)*” (North 1991, p. 97–112).

These institutions, clearly, have a life of their own, appearing and disappearing continuously (Acemoglu and Robinson 2012, Ch. 4). Thus, one way of understanding ‘institutions’ is in terms of a continuing set of adaptations, corrections and improvements on an earlier systemic pattern. In this sense, we may interpret the presence of systemic interest as being reflected in a continuous set of adaptations and re-structuring of existing structures, to protect the interest of those concerned. We refer here also to section 3 in which we have discussed the relationship between institutions and economic resilience.

Interestingly for our cases, this process can be followed over several decades in the case of three Dutch Caribbean island states Aruba, Curaçao, and Sint Maarten. Limiting ourselves to the essence, the most important changes are as given below.

## Exploring the Case of Aruba, Curaçao, and Sint Maarten

With the signing of the Charter of the Kingdom of the Netherlands (Dutch Kingdom) in 1954, the country Netherlands Antilles – consisting of Aruba, Bonaire, Curacao, Saba, Sint Eustatius, and Sint Maarten – obtained constitutional equality with the Netherlands, resulting in the end of the colonial rule. With this new status, this country was granted the authority to decide over its own domestic affairs. However, the Dutch Kingdom remained responsible for issues related to foreign affairs, defence, independence, and Dutch citizenship (Pereira 2018, p. 36). Noticeably, the safeguarding of fundamental human rights and freedoms, legal certainty and good governance is deemed a ‘Kingdom affair’, implying that the Dutch Caribbean islands are therefore de facto supervised by and where/when necessary corrected by the government of the Netherlands which has the majority of representatives in the Council of Ministers of the Kingdom (Nauta 2011, p. 24–26, p. 40–41).

In the 1960s and 1970s, the socio-economic discrepancies between the Netherlands and the Netherlands Antilles were increasing, resulting in a significant rise in the immigration from residents of the Netherlands Antilles to the Netherlands (Croes 2011, p. 20). Moreover, as a result of the political disturbances in Curaçao on May 30, 1969, the Netherlands was obliged because of its duties in accordance with the Charter of the Dutch Kingdom to conduct a military intervention in Curaçao in order to restore public order. These developments put the political independence of the Netherlands Antilles on the agenda of the Dutch politicians, because they felt that the Netherlands had too much obligations according to this Charter (Croes 2011, p. 26). This led in Aruba to discussions about its own status. Aruba was interested in a solution separated from the rest of the Netherlands Antilles (Giacalone 2001, p. 97). Aruba was granted the status of *Status Aparte* and thereby became an autonomous country within the Dutch Kingdom in 1986, but with a precondition that Aruba would obtain its independence in 1996. However, in the 1990s the Netherlands began to accept that self-determination did not necessary imply independence (Nauta 2011, p. 142), and a broad political consensus emerged that the Dutch Caribbean islands would be better off remaining part of the Dutch Kingdom (De Jong 2009, p. 27). Consequently, the Netherlands decided to let go the precondition of independence for Aruba.

Since the Status Aparte of Aruba, the viability of the Netherlands Antilles has been questioned (De Jong 2009, p. 30). Curaçao and Sint Maarten showed interest to obtain country status. In the end, the Netherlands Antilles was dissolved on October 10, 2010. At that time, Curaçao and Sint Maarten became autonomous countries within the Dutch Kingdom, while Bonaire, Saba, and Sint Eustatius became special municipalities of the Netherlands. Curacao and Sint Maarten had a different autonomous status as compared to Aruba, with regulation of public finances and law enforcement (De Jong 2009, p. 38). However, independent supervision of Aruba’s public finances by the Council of Ministers of the Dutch Kingdom became a reality in 2015.

One may argue that the relationship between on the one hand the Netherlands and on the other hand each of the countries Aruba, Curacao, and Sint Maarten is based on ‘systemic interest’ on both sides. From the point of view of the Netherlands, the following can explain its interest. In the first place, the Netherlands has a great interest in ensuring good governance in the Dutch Caribbean islands, because the (potential) noncompliance with this reflects not only on these countries but also on the Kingdom as a whole. From a viewpoint of reputational risk, the Netherlands definitely has an interest in ensuring good governance in these countries. In the second place, the problems of homicide and drug trafficking in the Dutch Caribbean also has affected the good reputation of the Dutch Kingdom, but they also spread through migration to the Netherlands (De Jong 2009, p. 32). In the third place, the high public debt level of the Netherlands Antilles contributed to the endeavour of the Netherlands to pursue good governance in that country (De Jong 2009, p. 32). This was also the case in Aruba, which was put under financial supervision of the Netherlands in 2015. In the fourth place, the presence of the Netherlands in the Caribbean has a value added for its allies, such as the United States, in combating drug trafficking and money laundering (Giacalone 2001, p. 98).

From the viewpoint of Aruba, Curaçao, and Sint Maarten many benefits exist for being part of the Dutch Kingdom. One important benefit is that being part of the Dutch Kingdom entails political stability, but has also resulted in financial aid from the Netherlands (Giacalone 2001, p. 98–99). Because of their status as overseas countries and territories (OCT) of the European Union, these countries have special access to the European and US markets, their residents can move freely in Europe, and they have benefited from aid from the European Community (Giacalone 2001, p. 98–99). But these islands could also benefit from economic relations of the Netherlands. For instance, Curaçao had benefited from economic agreements with the United States (Giacalone 2001, p. 98). This benefit relates to the quality of economic connectivity of the Netherlands which provides certain benefits for the Dutch Caribbean islands. One can think here of increased possibilities for foreign investments and also greater access to private foreign capital, but also of exports of services, such as tourism services in the case of Aruba. Another benefit of the ties with the Netherlands is that Aruba, Curaçao, and Sint Maarten can run their own affairs, thereby enjoying a most autonomous arrangement, while at the same time being part of the Dutch Kingdom; this compared to the British Overseas Territories (Ramos 2001, p. xvii).

In the decades following the enactment of the Charter of the Dutch Kingdom, several institutions and organizations were introduced based on the Dutch model, but also with the purpose of ensuring good governance. Although there was a huge difference between the *de jure* and de facto institutions in the (former) Netherlands Antilles (Nauta 2011, p. 153), through these decades, the Netherlands has pursued a convergence of good governance between itself and the islands. One example of this is associated with Sint Maarten. In response to a number of investigations related to integrity^10^ and following indications of the Netherlands, it signed a protocol in 2015, agreeing on the establishment of an independent Integrity Chamber for Sint Maarten. Following a delay on the part of Sint Maarten to establish this chamber, the Netherlands decided to put the approval of the law regarding the establishment of this chamber by the Parliament of Sint Maarten as one precondition for providing funds to Sint Maarten for its reconstruction after the damage caused by hurricane Irma in September 2017. Another example relates to the recent economic crisis in the Dutch Caribbean islands as a result of the Covid-19 pandemic. The Netherlands provided liquidity assistance to these countries, but it also demanded the countries to implement structural reforms, among others related to governance, to continue receiving this liquidity support. These examples illustrate the tendency for convergence of governance between the Netherlands and the Dutch Caribbean islands. This argument is supported by Oostindie and Sutton (2006) who argue that there seems to be a tendency towards convergence of good governance between dependent countries and territories and their relevant former colonizer.

Concluding, the relationship of the dependent small island states with their former colonizer may have led to better institutions, better governance, and economic connectivity of higher quality compared to the sovereign Caribbean small island states; this could have contributed to higher macroeconomic stability and economic resilience. We can understand the Dutch policy over time as an effort to assist the island states to stand to become fully financially and economically independent and not having to rely on the former colonizer. Compared to other sovereign Caribbean countries, we can mention that this policy points largely to a success, particularly in the case of Aruba. Nevertheless, ‘good governance’ in the Dutch Caribbean islands is not yet at the standards of the Netherlands. There remain many challenges in this area.

Thus, connectivity, when viewed in terms of systemic interest, can help us understand why the politically dependent countries and territories in the Caribbean may have higher economic resilience as compared to their sovereign peers. Therefore, it may be worthwhile to interpret the dynamics of the connection between the Netherlands and the Dutch Caribbean in a separate study, thereby primarily focusing on systemic interest on the parts of the stakeholders and parties involved.

## Concluding Remarks

In this paper we have examined the overall exposure of Caribbean small island states to exogenous shocks by using a holistic approach for measuring their economic vulnerability and resilience. Our point of departure was the new institutional economics as developed by Douglass North and others. Institutions are the ‘rules of the game’ and we may expect those countries to perform well that have made institutional choices that mitigate the consequences of their size. In particular, we have explored the relationship between political sovereignty (i.e., an institutional choice) and economic resilience by focusing on sovereign as well as dependent Caribbean small island states. We have looked at the role of connectivity of the dependent Caribbean small island states in explaining their above average economic resilience as compared to their sovereign peers.

Caribbean small island states are in general highly vulnerable to exogenous economic shocks, due largely to their small size constraints. Our estimates for both the degree of economic vulnerability and resilience among Caribbean small island states indicate that there is a diversity in the overall exposure to exogenous economic shocks. It seems that the smaller countries are more vulnerable than the larger countries, largely because of their dependence on foreign trade as reflected in their trade openness and export concentration. Undoubtedly, this is related to the combination of their small domestic market and demand for products that are not produced domestically. Nevertheless, despite their small size constraints, Caribbean small island states can survive economically, by building up their economic resilience by way of fortifying their institutions. Our results indicate that some countries are better equipped to deal with exogenous economic shocks than others. Clearly, these Caribbean small island states may have mitigated their economic disadvantages related to their size, because of their institutional choices. The latter may have contributed to their economic resilience. In fact, our results suggest that in general the dependent countries and territories have an edge above the sovereign states, possibly because of their ties with their former colonizer. Despite their high vulnerability, they have policy-induced resilience, in part as a result of the convergence between governance in these states and the Netherlands or United Kingdom as well as the fact that these states benefit from their connectivity with the latter countries. In this paper, we have briefly discussed the notion of systemic interest, i.e. a state of affairs where the major stakeholders are prepared to invest in good relations with the former colonizer. Our analysis has shown that the connection between the Netherlands and the Dutch Caribbean island states has resulted into a relatively good performance of these islands as a result of systemic interest in keeping the relationship viable. As pointed out, this is reflected in a good (or in some cases even excellent) international status in areas such as in finance, defense, or assistance in combating the impact of major catastrophes such as hurricanes and pandemics like COVID-19.

We should note that there are a number of limitations to our research. First, one of the limitations relates to limited data availability. As mentioned before, alternative data sources have been used to make the calculations, especially for the dependent Caribbean small island states. This may have impacted the accuracy of the calculations. Second, another limitation refers to the small sample size. Because of this, we have used relatively simple statistical tests such as a Mann-Whitney U-test. Third, we use simple weights for the economic vulnerability and economic resilience indices. This is in part because one of the biggest challenges is to get relevant data for these Caribbean small island states. Finally, we mention that future research may focus on constructing the composite indices based on different data weighting methods. Moreover, the economic resilience index could be amended to include variables that measure institutions and/or institutional quality more explicitly. Also, more research into the relationship between economic resilience and institutional choices in the Caribbean small island states will be necessary. As pointed out, the notion of systemic interest may play a central role here.

## Supplementary Information

ESM (DOCX 76 kb)

## Footnotes

1 In this paper, the term state is used to refer to both sovereign states and dependent countries and territories.

2 Following the definition of a small state, i.e., a country or territory with a population of 1.5 million or less, also the following Caribbean countries/territories should have been included in our study: Bonaire, British Virgin Islands, Guadeloupe, Martinique, Montserrat, Saba, Sint Eustatius, and Turks and Caicos Islands. However, because these islands are not included in the majority of databases used and no reliable alternative was found, we decided to exclude them from this study.

3 The Max-Min formula for rescaling or normalizing the value of an observation is the following: XR_i_ = (X_i_ – X_min_)/(X_max_ – X_min_) where X_i_: a particular observation i in an array of n observations X_max_: the maximum value in the same array of n observations X_min_: the minimum value in the same array of n observations XR_i_: the re-scaled (or normalized) observation i in the same array of n observations

4 A detailed discussion of data and data sources used is available online.

5 Due to the small sample size we use a nonparametric test, because we expect that we cannot test one of the underlying assumptions of its parametric equivalence. We refer here to the underlying assumption that the independent variable is normally distributed. This is because normality tests have little power to reject the null hypothesis of normality in the case of small sample size.

6 Please refer to footnote 5 for the reason we use this nonparametric test. For the sake of completeness, we note that applying the Pearson correlation yields the same results, i.e., a Pearson correlation of −0.681.

7 The average population size is 281 thousand. The log of this number is 2.449.

8 Please refer to footnote 5 for the reason we use this nonparametric test. For the sake of completeness, we note that applying the Pearson correlation yields the same results, i.e., a Pearson correlation of −0.191.

9 We use the guidelines given by Cohen (1988) to define the strength of the association. A correlation coefficient between −0.1 and − 0.3 is considered a weak association.

10 See General Audit Chamber (2014) and Commissie Integer Openbaar Bestuur (2014).
