# Supplementary material for: Vulnerability and Resilience in the Caribbean Island States; the Role of Connectivity
Source: Netw Spat Econ. 2021 May 27;22(3):515–40. doi: 10.1007/s11067-021-09533-w (PMC8159251; doi:10.1007/s11067-021-09533-w)
Supplement: Supplementary file 1 — ESM (DOCX 76 kb) [file 11067_2021_9533_MOESM1_ESM.docx]

**Vulnerability and resilience in the Caribbean island states;**

**The role of connectivity**

**Sources of data**

The Economic Vulnerability Index (EVI) of Briguglio (2014**)** covers the following four components, each with a weight of 25 percent in the overall EVI, because of their positive correlation to the exposure to external economic shocks:

1. Trade openness as estimated by the average of exports and imports of goods and services as percentage of GDP;
2. Export concentration as measured by the three highest export categories in total exports of goods and services;
3. Dependence on strategic imports as gauged by the imports of food and fuel as percentage of total merchandise imports;
4. Proneness to natural disasters as calculated in terms of economic damage relative to GDP.

EVI_i_ = 0.25 OPN_i_ + 0.25 EXN_i_ + 0.25 DSI_i_ + 0.25 DST_i_

EVI_RS_i_ = (EVI_i_ – EVI_min_)/(EVI_max_ – EVI_min_)

where

DSI_i_ : normalized value of dependence on strategic imports of country i

DST_i_ : normalized value of proneness to natural disasters of country i

EVI_i_ : economic vulnerability index for country i,

EVI_min_  : minimum value of EVI in the array of n countries

EVI_max_ : maximum value of EVI in the array of n countries

EVI_RS_i_ : re-scaled or normalized value of EVI for country i

EXN_i_ : normalized value of export concentration of country i

OPN_i_  : normalized value of trade openness of country i

Briguglio’s economic resilience index (ERI) includes the following three main components with equal weights of 33.33 percent:

1. Macroeconomic stability as measured by the average of its three subcomponents, i.e., government debt to GDP ratio, the inflation rate (measured as the GDP deflator), and the current account balance of the balance of payments as percentage of GDP;
2. Adjusted market flexibility as estimated by on the one hand the market flexibility index (weight of 25 percent in the overall ERI) and on the other hand the financial safety index (weight of 8.33 percent in the overall ERI)^[[1]](#footnote-1)^. The latter is calculated as the obverse of the financial riskiness index which combines the lack of financial prudence with the degree of financial depth;
3. And a political, social and environmental governance index as estimated by its three subcomponents, i.e., its political governance index (weight of 22.22 percent in the overall ERI), social development index (weight of 5.56 percent in the overall ERI), and the environmental management index (weight of 5.56 percent in the overall ERI)^[[2]](#footnote-2)^. The political governance index is assessed by the World Bank Worldwide Governance Indicators. The social development index is measured by the non-income components of the Human Development Index that relate to education and health, i.e., mean years of schooling, expected years of schooling and life expectancy at birth. The environmental management index is measured by the Environmental Performance Index.

ERI_i_ = 0.3333 STB_i__RS+ 0.25 MFX_i_ + 0.0833 FIN + 0.2222 PGV_i_ + 0.0566 SOC_i_ + 0.0566 ENV_i_

ERI_RS_i_ = (ERI_i_ – ERI_min_)/(ERI_max_ – ERI_min_)

STB_i_ = (DEBT_i_ + INF_i_ + CAB_i_) /3

STB_i__RS = (STB_i_ – STB_min_)/( STB_max_ – STB_min_)

where

CABi : normalized value of current account balance of the balance of payments of

country i

DEBTi : normalized value of government debt to GDP ratio of country i

ENVi : normalized value of environmental management index of country i

ERI_i_ : economic resilience index for country i,

ERI_max_ : maximum value of ERI in the array of n countries

ERI_min_  : minimum value of ERI in the array of n countries

ERI_RS_i_ : re-scaled or normalized value of ERI for country i

INFi : normalized value of inflation rate of country i

FIN_i_ : normalized value of financial safety index of country i

MFX_i_ : normalized value of market flexibility index of country i

PGVi : normalized value of political governance index of country i

SOCi : normalized value of social development index of country i

STB_i_  : macroeconomic stability of country i

STB_max_ : maximum value of STB in the array of n countries

STB_min_ : minimum value of STB in the array of n countries

STB_i__RS : normalized value of macroeconomic stability of country i

The sources of data for the (sub)components are given below.

1. *Trade openness*

Trade openness is measured by the average of exports and imports of goods and services as a percentage of GDP, averaged over three years (2014-2016^[[3]](#footnote-3)^).

Source: <http://unctadstat.unctad.org/>.

1. *Export concentration*

Export concentration is measured by the sum of the three broad groups of exports of goods and services which together have the highest share in total exports of goods and services. The data are averaged over the period 2015-2017.

The following product categories are distinguished:

1. Food and live animals
2. Beverages and tobacco
3. Crude materials, inedible, except fuels
4. Mineral fuels, lubricants and related materials
5. Animal and vegetable oils, fats and waxes
6. Chemicals and related products, not elsewhere specified (n.e.s.)
7. Manufactured goods
8. Machinery and transport equipment
9. Miscellaneous manufactured articles
10. Commodities and transactions, n.e.s.
11. Transport
12. Travel
13. Financial services
14. Other services

Source: <http://unctadstat.unctad.org/>.

Exceptions:

1. Aruba: Central Bureau of Statistics Aruba ([www.cbs.aw](http://www.cbs.aw)) for the breakdown of merchandise exports;
2. Curaçao: Central Bank of Curaçao and Sint Maarten ([www.centralbank.cw](http://www.centralbank.cw)) for the breakdown of merchandise exports;
3. Suriname: Central Bank of Suriname for the breakdown of merchandise exports (<https://www.cbvs.sr>).
4. *Dependence on strategic imports*

The dependence on strategic imports is measured by the imports of food^[[4]](#footnote-4)^ and fuel as a percentage of total merchandise imports. The data are averaged over the period 2015-2017.

Source: <http://unctadstat.unctad.org/>.

Exceptions:

1. Aruba: Central Bank of Aruba ([www.cbaruba.org](http://www.cbaruba.org)); Central Bureau of Statistics of Aruba ([www.cbs.aw](http://www.cbs.aw));
2. Cayman Islands: The Economics and Statistics Office (2018b);
3. Curaçao: Central Bank of Curaçao and Sint Maarten ([www.centralbank.cw](http://www.centralbank.cw)); Central Bureau of Statistics of Curaçao ([www.cbs.cw](http://www.cbs.cw)).
4. *Proneness to natural disasters*

The proneness to natural disasters is measured in terms of economic damage relative to GDP. The data are averaged over the period 1988-2017.

Source: EM-DAT database of the Université Catholique de Louvain, [www.emdat.be](http://www.emdat.be); UNDATA database for the GDP.

Exceptions:

1. Aruba: Meteorological Department of Curaçao (<http://www.meteo.cw>);
2. Curaçao: Meteorological Department of Curaçao (<http://www.meteo.cw>); GDP data prior to 2010 are derived from GDP of the Netherlands Antilles (UNDATA database);
3. Sint Maarten: estimates of the Netherlands Antilles are used for the years prior to 2010 (EM-DAT database). The share of Sint Maarten is largely calculated based on its GDP in the overall GDP of the Netherlands Antilles.
4. *Macroeconomic stability*

Macroeconomic stability is measured as the equally weighted average of the normalized values of three variables, i.e., government debt as a percentage of GDP, inflation measured by the GDP deflator, and current account balance in percentage of GDP. Since the first two variables are undesirables with regard to macroeconomic stability, the formula used for normalization of these variables is 1- [(X_i_ – X_min_)/(X_max_ – X_min_)]. The data are averaged over the period 2008-2017.

Source: IMF World Economic Outlook Database April 2019 and Database October 2017.

Exceptions:

1. Anguilla: Alleyne et al (2016); Eastern Caribbean Central Bank (<https://www.eccb-centralbank.org>); International Monetary Fund (2012);
2. Cayman Islands: Database The Economics and Statistics Office of the Cayman Islands (www.eso.ky); The Economics and Statistics Office (2012; 2014; 2015; 2016; 2017; 2018a).
3. Curaçao and Sint Maarten: Central Bureau of Statistics of the Netherlands Antilles ([www.cbs.cw](http://www.cbs.cw)); International Monetary Fund (2011; 2014; 2016b; 2019).
4. *Adjusted Market flexibility*

The adjusted market flexibility index is a combination of the market flexibility index and the financial safety index.

1. *Market flexibility index*

We use the variable ‘Distance to frontier’ (DTF) from the World Bank’s Doing Business Index (DBI) instead of the Economic Freedom of the World Index used by Briguglio (2014). The DTF assesses the absolute level of regulatory performance over time. The frontier is defined as the best performance observed on each of the indicators of the DBI across all economies included in the database of the DBI. An economy’s DTF is reflected on a scale from 0 to 100 where 0 represents the lowest performance and 100 represents the frontier. The data are averaged over the period 2015-2017.

Source: <http://www.doingbusiness.org/data/distance-to-frontier>.

Exceptions:

Anguilla, Aruba, Cayman Islands, Curaçao, and Sint Maarten: These countries are not included in the DTF-database. Therefore, the Regulatory Quality indicator of the Worldwide Governance Indicators of the World Bank is explored. For Curaçao and Sint Maarten, the Regulatory Quality data of the Netherlands Antilles for the years 2011-2013 are used, because of lack of data for these countries. The Regulatory Quality indicator assesses the ability of the government to formulate and implement sound policies and regulations that permit and promote private sector development. Mentioned countries with missing data for DTF have the highest scores for the Regulatory Quality indicator compared to the selected countries that are included in the DTF. This suggests that Anguilla, Aruba, Cayman Islands, Curaçao, and Sint Maarten have a high score for market flexibility compared to the other selected countries. Nevertheless, to be on the safe side, we assume that the DTF scores for Anguilla, Aruba, Cayman Islands, Curaçao, and Sint Maarten are equal to the median of the other 12 selected Caribbean countries and territories.

1. *Financial safety index*

Our estimate of the financial safety index is composed of a financial prudence index weighted by the importance of the financial sector. Consequently, it differs from the index calculated by Briguglio (2014). He estimates a financial safety index as the obverse of the financial riskiness index which combines the lack of financial prudence with degree of financial depth.

The data for estimating our financial safety index and its components are averaged over the period 2013-2017.

The financial prudence component of our financial safety index is estimated as the equally weighted average of a ‘soundness of banks’ index and a ‘strength of legal rights’ (SOLR) index.

The soundness of banks index comprises four variables: regulatory capital to risk weighted assets (in percent), non-performing loans to total gross loans (in percent), return on assets (in percent), and liquid assets to total assets (in percent). Note that data for the period of 2014-2017 are used in the case of Cayman Islands. Due to lack of data on the regulatory capital to risk weighted assets, the soundness of banks index for Dominica is based on the remaining three indicators. Since the variable ‘non-performing loans to total gross loans’ is undesirable with regard to soundness of banks, the formula used for normalizing this variable is 1-[(X_i_ – X_min_)/(X_max_ – X_min_)].

The World Bank’s SOLR measures the degree to which collateral and bankruptcy laws protect the rights of borrowers and lenders and thus facilitate lending. Anguilla, Aruba, Cayman Islands, Curaçao, and Sint Maarten are not included in the SOLR database. Based on the ordinary least squares method, a regression between SOLR and the Rule of Law (RoL) indicator of the Worldwide Governance Indicators of the World Bank is estimated to impute the SOLR for the countries with missing data. The RoL captures perceptions of the extent to which agents have confidence in and abide by the rules of society, and in particular the quality of contract enforcement, property rights, the police, and the courts, as well as the likelihood of crime and violence.

Mentioned regression is based on data for the period 2013-2017 for 187 countries with available data in the SOLR-database.

The estimated equation is:

SOLR = 5.1 + 0.8 RoL

*t-stat 24.595 3.671*

R^2^ = 0.068; N= 187

The importance of the financial sector is measured as the log of the domestic credit to private sector by banks (% of GDP), to account for the diminishing marginal effect of the size of the financial sector.

Sources:

1. Financial soundness indicators: Bank of Guyana (<http://www.bankofguyana.org>); Cayman Islands Monetary Authority (2017a; 2017b; 2018); Central Bank of Barbados (n.d.); Central Bank of Belize (<https://www.centralbank.org.bz>); Centrale Bank van Aruba (2018); Eastern Caribbean Central Bank (<https://www.eccb-centralbank.org>); Financial soundness indicators database of the IMF ([http://data.imf.org/?sk=51B096FA-2CD2-40C2-8D09-699CC1764DA&sId =1390030341854](http://data.imf.org/?sk=51B096FA-2CD2-40C2-8D09-699CC1764DA&sId%20=1390030341854)); International Monetary Fund (2018a; 2018c; 2018d; 2019); World Bank (2018);
2. SOLR: <https://data.worldbank.org/indicator/ic.lgl.cred.xq>;
3. RoL: <https://data.worldbank.org/data-catalog/worldwide-governance-indicators>; In the case of Curaçao and Sint Maarten, the 2013 data for the Netherlands Antilles are used;
4. Domestic credit to private sector: Cayman Islands Monetary Authority (2017b); Eastern Caribbean Central Bank (<https://www.eccb-centralbank.org>); International Monetary Fund (2016a; 2018b; 2019), World Bank (<https://data.worldbank.org/data-catalog/world-development-indicators>). Note that for Curaçao and Sint Maarten the consolidated data for the monetary union of those two countries are used.
5. *Political Governance*

Political governance is measured as the equally weighted average of the six Worldwide Governance Indicators of the World Bank, i.e., voice and accountability, political stability and absence of violence, government effectiveness, regulatory quality, rule of law, and control of corruption.

Data for the period 2015-2017 (average) are used.

Source: <https://data.worldbank.org/data-catalog/worldwide-governance-indicators>.

Exceptions:

For Curaçao and Sint Maarten, the Worldwide Governance Indicators of the Netherlands Antilles for the years 2011-2013 are used, because these islands were part of the Netherlands Antilles until October 10, 2010. On the latter date, the country Netherlands Antilles was dissolved. Data for the period 2015-2017 for these two countries are not available.

1. *Social Development*

Social development is measured as the equally weighted average of the normalized values of the three components of the Human Development Index, i.e., mean years of schooling, expected years of schooling and life expectancy at birth. There are missing data for a number of countries, i.e., Anguilla, Sint Maarten.

Data for the period 2015-2017 (average) are used.

Source: <http://hdr.undp.org/en/data>.

Exceptions:

1. Anguilla: The mean years of schooling and the expected years of schooling are estimated to be equal to the median for all other countries for which data are available. The life expectancy at birth is equal to the available 2013 data (source: [https://www.paho.org/](https://www.paho.org/salud-en-las-americas-2017/?page_id=79%20Retrieved%2012%20June%202019) );
2. Aruba: The expected years of schooling is equal to the average of 2010-2012 and the mean years of schooling is equal to year 2010 (source: <http://data.uis.unesco.org/>). Data for life expectancy (World Bank World Development Indicators);
3. Cayman Islands: The mean years of schooling is equal to the average of 2014-2015 (source: <http://data.uis.unesco.org/>), while the expected years of schooling is derived from the mean years of schooling by multiplying the average ratio of expected years of schooling to mean years of schooling of other countries with the mean years of schooling of Cayman Islands. Data for life expectancy for the year 2010 (World Bank World Development Indicators);
4. Curaçao: The mean years of schooling is equal to the year 2012 and the expected years of schooling is equal to year 2013 (source: <http://data.uis.unesco.org/>). Data for life expectancy (World Bank World Development Indicators);
5. Sint Maarten: The mean years of schooling and the expected years of schooling are estimated to be equal to the median for all other countries for which data are available. Data for life expectancy for the year 2012 (World Bank World Development Indicators).
6. *Environmental Management*

The Environmental Management index is based on the Environment Performance Index (EPI) which is produced jointly by Yale University and Columbia University in collaboration with the World Economic Forum and is available at <http://epi.yale.edu/>. The 2018 EPI ranks 180 countries on 24 performance indicators across ten issue categories covering environmental health and ecosystem vitality. It therefore provides a gauge at a national scale of how close countries are to established environmental policy goals. The EPI of 2018 is used.

Source: <https://epi.envirocenter.yale.edu/2018/report/category/hlt>.

For countries with missing data in the EPI database – specifically Anguilla, Aruba, Cayman Islands, Curaçao, Grenada, Sint Maarten, and Saint Kitts & Nevis – an ordinary least squares multiple regression with the following explanatory variables is estimated to impute an EPI-score for these countries:

1. GDP per capita (denoted as GDP_PC; source: UNDATA database; average of 2015-2017);
2. Government Effectiveness indicator of the Worldwide Governance Indicators of the World Bank (denoted as GovEff; source: World Bank Worldwide Governance Indicators; average of 2015-2017);
3. Share of Agriculture, Hunting, Forestry and Fishing in GDP (denoted as AHFF, source: UNCTAD database; average 2015-2017);
4. The subcomponent “Terrestrial Protected Areas (Global Weights) / Biodiversity & Habitat / Ecosystem Vitality” of the EPI (denoted as TBG; source: <https://epi.envirocenter.yale.edu/2018/report/category/hlt>; average of 2015-2017).

The estimated equation is based on data of 171 countries:

EPI = 51.3 + 0.0002 GDP_PC + 4.1 GovEff + -0.3AHFF + 0.5 TBG

*t-stat 33.646 4.631 4.003 -5.946 5.427*

R^2^ = 0.733; N= 171

**References**

Alleyne D, Hendrickson M, McLean S et al (2016) Economic Survey of the Caribbean 2015: Balancing economic recovery and high debt in the Caribbean. ECLAC Studies and Perspectives 50. <https://repositorio.cepal.org/bitstream/handle/11362/39857/1/S1501385_en.pdf>

Briguglio L (2014) A vulnerability and resilience framework for small states. Report prepared for The Commonwealth Secretariat.

<https://www.um.edu.mt/library/oar/bitstream/123456789/18015/1/Chapter%20-%20%20A%20Vulnerability%20and%20Resilience%20Framework%20for%20Small%20States.pdf>

Cayman Islands Monetary Authority (2017a) Banking sector statistical digest and prudential surveillance report 2015. <https://www.cima.ky/upimages/publicationdoc/BankingStatistical_1499166484.pdf>

Cayman Islands Monetary Authority (2017b) Banking statistical digest 2016.

<https://www.cima.ky/upimages/publicationdoc/BankingStatistical_1513711868.pdf>

Cayman Islands Monetary Authority (2018) Banking statistical digest 2017. <https://www.cima.ky/upimages/publicationdoc/BankingStatistical_1540404146.pdf>

Central Bank of Barbados (n.d.) Financial stability report 2017.

<http://www.centralbank.org.bb/Portals/0/2017Financial%20Stability%20Report.pdf>

Centrale Bank van Aruba (2018) Financial sector supervision report 2017.

<https://www.cbaruba.org/cba/readBlob.do?id=4717>

International Monetary Fund (2011) Kingdom of the Netherlands—Curaçao and Sint Maarten: 2011 Article IV consultation—Staff report; Informational annex; and Public information notice on the Executive Board discussion. IMF Country Report No. 11/342.

<https://www.imf.org/external/pubs/ft/scr/2011/cr11342.pdf>

International Monetary Fund (2012) United Kingdom—Anguilla— British Overseas Territory: 2011 Article IV consultation discussions. IMF Country Report No. 12/8.

<https://www.imf.org/en/Publications/CR/Issues/2016/12/31/United-Kingdom-Anguilla-British-Overseas-Territory-Staff-Report-for-the-2011-Article-IV-25659>

International Monetary Fund (2014) Kingdom of The Netherlands— Curaçao and Sint Maarten: 2014 Article IV consultation discussions—Staff report and press release. IMF Country Report No. 14/239.

<https://www.imf.org/en/Publications/CR/Issues/2016/12/31/Kingdom-of-the-Netherlands-Curaçao-and-Sint-Maarten-2014-Article-IV-Consultation-Staff-41809>

International Monetary Fund (2016a) Barbados 2016 Article IV consultation—Press release; Staff report; and statement by the executive director for Barbados. IMF Country Report No. 16/279.

<https://www.imf.org/en/Publications/CR/Issues/2016/12/31/Barbados-2016-Article-IV-Consultation-Press-Release-Staff-Report-and-Statement-by-the-44205>

International Monetary Fund (2016b) Kingdom of The Netherlands–– Curaçao and Sint Maarten:

2016 Article IV consultation discussions— Press release; Staff report and informational annex. IMF Country Report No. 16/276.

<https://www.imf.org/en/Publications/CR/Issues/2016/12/31/Kingdom-of-the-Netherlands-Curaçao-and-Sint-Maarten-2016-Article-IV-Consultation-Discussions-44194>

International Monetary Fund (2018a) Bahamas 2018 Article IV consultation—Press release and staff report. IMF Country Report No. 18/118.

<https://www.imf.org/en/Publications/CR/Issues/2018/05/14/The-Bahamas-2018-Article-IV-Consultation-Press-Release-and-Staff-Report-45874>

International Monetary Fund (2018b) Barbados 2017 Article IV consultation—Press release; Staff report; and statement by the executive director for Barbados. IMF Country Report No. 18/133.

<https://www.imf.org/en/Publications/CR/Issues/2018/05/30/Barbados-2017-Article-IV-Consultation-Press-Release-Staff-Report-and-Statement-by-the-45913>

International Monetary Fund (2018c) Guyana 2018 Article IV consultation—Press release and staff report. IMF Country report no. 18/220.

<https://www.imf.org/en/Publications/CR/Issues/2018/07/16/Guyana-2018-Article-IV-Consultation-Press-Release-and-Staff-Report-46083>

International Monetary Fund (2018d) Suriname 2018 Article IV consultation discussions; Press release; Staff report and statement by the executive director for Suriname. IMF Country report No. 18/376.

<https://www.imf.org/en/Publications/CR/Issues/2018/12/20/Suriname-2018-Article-IV-Consultation-Press-Release-Staff-Report-and-Statement-by-the-46487>

International Monetary Fund (2019) Kingdom of The Netherlands—Curaçao and Sint Maarten:

2018 Article IV consultation discussions— Press release and staff report. MF Country Report No. 19/23.

<https://www.imf.org/en/Publications/CR/Issues/2019/01/25/Kingdom-of-the-Netherlands-Curaao-and-Sint-Maarten-2018-Article-IV-Consultation-Press-46543>

The Economics and Statistics Office (of Cayman Islands) (2012) Annual economic report 2011.

<https://www.eso.ky/UserFiles/page_docums/files/uploads/docum444.pdf>

The Economics and Statistics Office (of Cayman Islands) (2014) Annual economic report 2013.

<https://www.eso.ky/UserFiles/page_docums/files/uploads/cayman_islands_annual_economic_report_20-1.pdf>

The Economics and Statistics Office (of Cayman Islands) (2015) Annual economic report 2014.

<https://www.eso.ky/UserFiles/page_docums/files/uploads/the_cayman_islands__annual_economic_repo-1.pdf>

The Economics and Statistics Office (of Cayman Islands) (2016) Annual economic report 2015.

<https://www.eso.ky/UserFiles/page_docums/files/uploads/the_cayman_islands__annual_economic_repo-2.pdf>

The Economics and Statistics Office (of Cayman Islands) (2017) Annual economic report 2016.

<https://www.eso.ky/UserFiles/page_docums/files/uploads/tha_cayman_islands_annual_economic_repor-2.pdf>

The Economics and Statistics Office (of Cayman Islands) (2018a) Annual economic report 2017.

<https://www.eso.ky/UserFiles/page_docums/files/uploads/the_cayman_islands_annual_economic_repor.pdf>

The Economics and Statistics Office (of Cayman Islands) (2018b) The Cayman Islands’ foreign trade statistics report 2017. <https://www.eso.ky/UserFiles/page_docums/files/uploads/the_cayman_islands_annual_foreign_trade.pdf>

World Bank (2018) International Development Association program document for a proposed programmatic development policy credit in the amount of SDR 24.4 million (US$35 million equivalent) to the Co-operative Republic Of Guyana for the first programmatic financial and fiscal stability development policy credit (p165425).

<http://documents.worldbank.org/curated/en/489761528122701470/pdf/Guyana-PD-06012018.pdf>

1. Briguglio (2014) provides no explanation for the choice of the weights for the subcomponents market flexibility index and financial safety index. He notes that several weighting schemes were tested besides the selected weighting scheme. [↑](#footnote-ref-1)
2. Briguglio (2014) gives no explanation for the choice of the weights for the subcomponents of the political, social and environmental governance index. He notes that several weighting schemes were tested besides the selected weighting scheme. [↑](#footnote-ref-2)
3. The data for this indicator for the year 2017 are not available for the majority of countries in the UNCTAD database. Therefore, the average for the period 2014-2016 is used. [↑](#footnote-ref-3)
4. All food items (SITC 0 + 1 + 22 + 4) from the database of UNCTAD. [↑](#footnote-ref-4)
